# Timescale and colony-dependent relationships between environmental conditions and plasma oxidative markers in a long-lived bat species

**DOI:** 10.1093/conphys/coaa083

**Published:** 2020-09-14

**Authors:** Michaël Beaulieu, Frédéric Touzalin, Serena E Dool, Emma C Teeling, Sébastien J Puechmaille

**Affiliations:** Zoological Institute & Museum, University of Greifswald, Loitzer Str. 26, 17489 Greifswald, Germany; German Oceanographic Museum, Katharinenberg 14-20, 18439 Stralsund, Germany; School of Biology and Environmental Sciences, University College Dublin, Belfield, Dublin 4, Ireland; Zoological Institute & Museum, University of Greifswald, Loitzer Str. 26, 17489 Greifswald, Germany; School of Biology and Environmental Sciences, University College Dublin, Belfield, Dublin 4, Ireland; School of Biology and Environmental Sciences, University College Dublin, Belfield, Dublin 4, Ireland; Zoological Institute & Museum, University of Greifswald, Loitzer Str. 26, 17489 Greifswald, Germany; School of Biology and Environmental Sciences, University College Dublin, Belfield, Dublin 4, Ireland; ISEM, University of Montpellier, CNRS, EPHE, IRD, Montpellier, France

**Keywords:** Bats, biomarkers, oxidative status, temperature anomalies

## Abstract

To increase the applicability and success of physiological approaches in conservation plans, conservation physiology should be based on ecologically relevant relationships between physiological markers and environmental variation that can only be obtained from wild populations. Given their integrative and multifaceted aspects, markers of oxidative status have recently been considered in conservation physiology, but still need to be validated across environmental conditions and locations. Here, we examined whether inter-annual variation in two oxidative markers, plasma antioxidant capacity and plasma hydroperoxides, followed inter-annual variation in temperature anomalies and associated vegetation changes in four colonies of long-lived greater mouse-eared bats (*Myotis myotis*) monitored over five consecutive years. We found that the plasma antioxidant capacity of bats decreased while plasma hydroperoxide concentrations increased with increasing temperature anomalies occurring in the two weeks before blood sampling. Moreover, the antioxidant defences of these bats reflected vegetation indices, which themselves reflected the thermal conditions experienced by bats in their foraging habitat. Variation in oxidative markers therefore appears to be due to variation in thermoregulatory costs and to indirect changes in foraging costs. Overall, these results validate the use of markers of oxidative status in conservation physiology to monitor thermal perturbations recently experienced by animals in their natural habitat. However, even though oxidative markers varied in the same direction in all four bat colonies across years, the amplitude of their response differed. If these different physiological responses reflect different performances (e.g. productivity, survival rate) between colonies, this implies that, if necessary, conservation measures may need to be applied at the local scale.

## Teaser

Physiological markers can be used in conservation studies if they reliably reflect environmental perturbations. Here, two oxidative markers reflected environmental conditions experienced by greater mouse-eared bats. However, the magnitude of the variation in oxidative markers differed among nearby colonies, suggesting that, if necessary, a one-size-fits-all conservation approach would be inappropriate.

## Introduction

Conservation physiology is based on the assumption that physiological markers reliably reflect environmental perturbations experienced by animals and can therefore be used to inform conservation plans for threatened animal populations (Cooke *et al.*, 2013). For instance, in the context of climate change, physiological markers can be used to track a *posteriori* the effects of novel environmental factors (e.g. long heatwaves, low food availability) on an animal’s condition, thereby allowing conservation practitioners to better characterize the threat that such novel conditions represent. Moreover, physiological parameters can be used to predict whether animals with given physiological characteristics will be able to sustain future environmental changes. Most of our knowledge on the relationship between physiological markers and environmental conditions is derived from studies conducted on animals in captivity. The results of these studies may be transposable to wild populations on the condition that the genetic pool of captive animals reflects the genetic pool of wild animals and that captive animals experience conditions comparable to those experienced in the wild. However, replicating all these conditions in captivity is logistically impossible. Studies conducted in captivity, therefore, typically only include animals coming from selected genetic lines able to acclimate to captivity (i.e. a limited genetic pool unlikely to be representative of the genetic pool in the wild). Moreover, these captive animals typically have ad libitum access to feeding resources (thereby potentially preventing resource allocation between physiological functions), are maintained under stable husbandry conditions (not necessarily representative of natural conditions) and experience a narrow range of environmental conditions when being experimentally tested (Beaulieu, 2016; Forstmeier *et al.*, 2007; Ricklefs and Cadena, 2007). For all of these reasons, the relationship between environmental conditions and physiological markers measured in captive animals may not necessarily be transposable to wild animals, and thus may hinder the success of conservation physiology approaches in the wild. Therefore, there is a need for data to be collected from wild animal populations experiencing ecologically relevant conditions, if we are to successfully implement the use of physiological markers in future conservation plans.

In conservation physiology, markers of oxidative status are used less frequently than other markers, such as immune markers and glucocorticoids (Beaulieu and Costantini, 2014; Madliger *et al.*, 2018). This is unfortunate given the integrative and multifaceted aspect of these physiological markers (Beaulieu and Costantini, 2014). Indeed, the oxidative status of animals can be assessed by measuring a variety of antioxidant defences (*e.g.* superoxide dismutase, catalase, glutathione) mobilized to counteract the damaging action of reactive oxygen species (ROS) mostly produced by mitochondria during aerobic energy production. By inhibiting or quenching ROS, antioxidant defences reduce the generation of oxidative damage on biomolecules such as lipids (e.g. malondialdehyde), proteins (e.g. protein carbonyls) or nucleic acids (e.g. 8-hydroxyguanosine). Because ROS production may vary following most environmental changes affecting the metabolism of organisms (e.g. pollution, thermal stress, dehydration), markers of antioxidant defences and oxidative damage (or at least some of them) should reflect specific environmental conditions experienced by animals (Beaulieu and Costantini, 2014). For instance, endotherms exposed to temperatures outside their thermoneutral zone (i.e. the range of temperatures for which thermoregulatory costs are minimal) elevate their metabolism to maintain a constant body temperature, thereby increasing ROS production and potentially some markers of oxidative damage (Al-Azraqi, 2008; Blagojević, 2007; Costantini *et al.*, 2012; Lin *et al.*, 2008). Moreover, a decline in prey availability, such as insects, due to cold or hot weather conditions (Bale *et al.*, 2002; Hallmann *et al.*, 2017; Lister and Garcia, 2018) may have similar effects on the oxidative status of predators by increasing foraging costs due to increased search time and muscular effort (Costantini *et al.*, 2008). In both cases (increased thermoregulatory costs and increased foraging costs), increased oxidative damage may be used as an indicator of thermally challenging conditions experienced by animals. It is, however, difficult to predict which molecules among all those involved in the regulation of the oxidative status will vary in response to given environmental conditions, as our understanding of which oxidative markers (e.g. enzymatic vs. non-enzymatic antioxidant defences) respond to given oxidative challenges is limited (Beaulieu and Costantini, 2014). The probability of finding oxidative markers able to track specific environmental conditions will therefore increase with the number of oxidative markers under investigation. Moreover, the oxidative status of animals is typically assumed to vary almost simultaneously with environmental conditions (Beaulieu and Costantini, 2014). However, depending on the kinetics and efficiency of the antioxidant response combined with the turnover rate of the tissue under consideration, variation in oxidative status may persist several days or weeks after exposure to oxidatively challenging conditions (e.g. increased physical activity, exposure to temperatures outside the thermoneutral zone; Ascensão *et al.*, 2008; Stambuk *et al.*, 2014; Rao *et al.*, 2014). The oxidative status of animals measured at a given time may therefore not necessarily reflect current conditions but the conditions animals previously experienced.

Consistent variation in physiological markers in relation to environmental conditions is an important pre-requisite to validate whether a given parameter can be used for conservation purposes (Madliger and Love, 2014). However, the antioxidant response and the resulting levels of oxidative damage may vary between individuals depending on a variety of factors. For instance, animals may respond differently to oxidatively challenging conditions because of the genetic basis of antioxidant defences (Costantini and Dell’Omo, 2006). At the population level, such inter-individual variation may lead to gene-environment interactions when variation in oxidative markers across environmental conditions differs between populations (Nussey *et al.*, 2007). Moreover, oxidative markers depend on the life-history strategies adopted by animals and reflect how animals prioritize life functions. For instance, higher antioxidant defences are expected in animals prioritizing self-maintenance over other functions (e.g. growth, reproduction; as expected in species following a *K*-selection strategy, such as long-lived species). In contrast, higher oxidative damage is expected in animals sacrificing self-maintenance for these functions (as expected in species following an *r*-selection strategy, such as short-lived species; Monaghan *et al.*, 2009). Such prioritizations are more likely to occur when resources are limited and need to be allocated between physiological functions (e.g. reproduction vs. survival; Beaulieu *et al.*, 2015).

Here, we investigated whether plasma oxidative markers reflected the thermal conditions experienced by free-ranging greater mouse-eared bats (*Myotis myotis*) by monitoring four geographically close maternity colonies in northwestern France across five consecutive years. *Myotis myotis* can live up to 37 years (Gaisler *et al.*, 2003) and their thermoneutral zone lies between a lower critical temperature of 28°C and an upper critical temperature of >37°C (Speakman, 2003), suggesting that they typically experience environmental temperatures below their thermoneutral zone when outside their roost. Depending on the amplitude of current climate warming experienced by bats from our investigated colonies, higher temperatures may expose them either to temperatures closer to their thermoneutral range (thereby reducing thermoregulatory costs) or to temperatures beyond their upper critical temperature (thereby increasing thermoregulatory costs). Moreover, a small temperature increase should result in higher insect abundance (thereby reducing the foraging costs of these insectivorous bats; Bale *et al.*, 2002; Hallmann *et al.*, 2017) while a strong temperature increase should result in reduced insect abundance (thereby increasing foraging costs; Lister and Garcia, 2018). Overall, increasing temperatures should represent an oxidative challenge due to higher thermoregulatory and foraging costs only if this temperature increase exposes bats to temperatures higher than their upper critical temperatures and/or to poor foraging conditions. Under this scenario, we expected long-lived reproductive bats to solve the trade-off between maintenance and reproduction by allocating resources to self-maintenance mechanisms such as antioxidant defences. Such resource reallocation may, however, still result in higher levels of oxidative damage if the antioxidant response is insufficient because of limited resources allocated to self-maintenance. Under the opposite scenario with only moderately higher temperatures associated with lower thermoregulatory and foraging costs, we expected bats to reduce their use of antioxidant defences due to lower ROS production. Oxidative damage may still remain high, as bats may in that case invest more into other functions, such as reproduction. Given the close proximity of the four considered colonies (and gene flow between colonies), we also expected all colonies to respond similarly to thermal conditions.

## Methods

### Animals

Bats were captured with custom harp traps when they were leaving their roost for the night to forage during the first two weeks of July between 2014 and 2018 at four locations in the South-East of the department of Morbihan, Brittany, France: La Roche-Bernard (47°31′08”N, 02°17′51”W), Férel (47°28′59”N, 02°20′33”W), Noyal-Muzillac (47°35′30”N, 02°27′25”W), and Béganne (47°35′49”N, 02°14′20”W). The area is an agricultural bocage (i.e. hedged farmland) region with an oceanic climate typical of northwestern France. Colonies were within 5–16 km from each other (mean ± SD, 12.6 ± 4.3 km). Bats were captured at each location every year, except bats from Béganne, which were sampled in 2015, 2017 and 2018. Blood (< 100 μl; < 5% of bats’ blood volume (7–10 ml); Neuweiler, 2000) was collected with heparinized capillaries after puncturing the uropatagial vein with sterile needles (26 gauge; as detailed in Huang *et al.*, 2016). Animals were weighed (± 0.1 g) and their body mass was used as an index of body condition and fat reserves (McGuire *et al.*, 2018), which may be related to the measures of lipid peroxidation we made to assess oxidative damage (see below; Pérez-Rodríguez *et al.*, 2015). Blood samples were kept on ice for ca. three hours before being centrifuged to separate plasma and red blood cells and stored at −80°C. Individuals were aged and sexed based on physical traits (e.g. swollen nipples in lactating adult females). We distinguished adult and juvenile bats based on the ossification of the epiphyseal cartilage of their metacarpal-phalangeal joints, which are fused in adults and unfused in juveniles (Kunz and Anthony, 1982). A total of 183 adult females, 35 adult males, 34 juvenile females and 42 juvenile males were captured and sampled. All juveniles and any unmarked adults were subcutaneously implanted with microtransponders (ID-100A, Trovan. Ltd, UK) in the back with sterile needles to allow future individual identification. For further analyses, we only considered adult females, for which we had a sufficient sample size.

To assess whether our chosen markers of oxidative status (see below) reflected baseline levels and not acute levels due to capture, we conducted a blood sampling experiment in 2015 to test whether retention duration (the time captured bats waited before blood sampling) influenced our results. Towards this end, eight adult females from an additional nearby colony (Saint-Nolff; 47°42′13”N, 02°39′07”W) were sampled twice: immediately upon capture and once again after 46–96 minutes of retention in a bag (mean ± SD, 77.2 ± 16.2 min). To avoid potential effects of sampling time on oxidative markers (van de Crommenacker *et al.*, 2011; Jenni-Eiermann *et al.*, 2014), all bats included in this experimental sampling were captured at the same time of night (from 23:05 to 23:33).

### Regional thermal conditions

Regional inter-annual thermal conditions were retrieved from Infoclimat.fr (version 5.4), which provided data collected by a permanent Météo-France weather station in Saint-Nazaire-Montoir (47°18′38”N, 02°09′24”W) located 30 ± 7 km (mean ± SD) from our four sampling locations. Daily minimal and maximal temperatures as well as temperature anomalies for minimal and maximal temperatures were provided. Temperature anomalies were calculated for both minimal and maximal temperatures as the difference between temperatures measured each day and reference monthly minimal and maximal temperatures averaged over the period 1981–2010 (as provided by InfoClimat; in July: minimal temperature is 14.0°C, maximal temperature is 24.4°C). This 30-year time-window is relevant for the bats used in our study, as, even though we did not know their precise age, bats sampled between 2014 and 2018 were born, grew and lived within this period of time.

### Local thermal and vegetation conditions

To examine whether colonies differed in their local thermal environments, we examined Land Surface Temperatures (LSTs; i.e. the radiative surface temperature) provided by the imaging sensor MODIS (Moderate Resolution Imaging Spectroradiometer; MOD11A2) on board the NASA satellite Terra. MODIS provides averaged 8-day per-pixel LST values with a spatial resolution of one kilometer. LST data were retrieved from Google Earth Engine (Gorelick *et al.*, 2017) every eight days between 26 June and 20 July for each of our study years. Mean LST values were calculated within a 1-km radius around each colony during the day, and within a 10-km radius around each colony at night. We chose these radii based on the fact that *M. myotis* bats remain in their roost during the day (1 km being the finest satellite resolution available) while they can forage 10 km away from their colony at night (Zahn *et al.*, 2006). Only images with a full coverage of the focal areas (i.e. with a clear sky) were used for further analyses.

We examined Normalized Difference Vegetation Index (NDVI) and Enhanced Vegetation Index (EVI) to quantify the green vegetation around each colony (Pettorelli *et al.*, 2011). Green vegetation was used as an ecological index potentially reflecting the quality of bats’ habitat, as greener vegetation may be more favourable to insects, with EVI values being a better predictor of insect abundance than NDVI in western France (Lafage *et al.*, 2014). NDVI and EVI values were retrieved from Google Earth Engine (Gorelick *et al.*, 2017), which provides data collected by the Earth observation satellite Landsat 8 with a 30 m resolution. NDVI values are calculated from the visible and near infrared light reflected by vegetation (NDVI = (reflectance near infrared − reflectance red)/(reflectance near infrared + reflectance red)) and range from −1 to +1 with higher values being associated with greener areas. EVI values are similar to NDVI values except that they correct for atmospheric conditions (through coefficients C of atmospheric resistance and values B from the blue band) and canopy background noise (through an L value). EVI values are calculated with the following formula: EVI = G * ((reflectance near infrared—reflectance red)/(reflectance near infrared + C1 x reflectance red—C2 x B + L)) with G = 2.5, C1 = 6, C2 = 7.5 and L = 1 in Landsat 8. As Landsat 8 measured NDVI and EVI values every eight days in a given region and because some of these days were cloudy, we considered NDVI and EVI values over a broader timescale (10 June–20 July) than the sampling period in each year. One to three cloud-free images were available within this time window each year for each colony (values were averaged into a single mean value when several images were available). Similar to night LST, we also considered a 10-km radius around each colony for vegetation indices. Average NDVI and EVI values were provided by Google Earth Engine within this 10-km radius area for cloud-free images. Finally, night LST values were also retrieved for the same dates as vegetation indices to examine the relationship between LST and vegetation indices within the same geographic areas.

An overview of the regional and local environmental parameters considered in our study is provided in Table 1.

**Table 1:** Environmental parameters measured in our study. Spatial and temporal information on measurements is provided as well as their relationship with the physiological parameters considered in our study. LST: Land Surface Temperature, NDVI: Normalized Difference Vegetation Index, EVI: Enhanced Vegetation Index.

Parameter			Spatial information	Temporal information	Correlation with physiological parameters
Regional conditions	Previous temperature anomalies Current temperature anomalies		Measured 30 ± 7 km from colonies	Daily measurement during sampling period Daily measurement in 2 weeks preceding sampling	− Antioxidant capacity + Hydroperoxides + Body mass
Local conditions	LST Vegetation indices	Day LST Night LST NVDI EVI	1-km radius around each colony 10-km radius around each colony 10-km radius around each colony	Measurement averaged over the previous 8 days (26 June–20 July) Measurement every 8 days (10 June–20 July)	+ Hydroperoxides − Antioxidant capacity + Antioxidant capacity

### Oxidative status

We measured two markers of oxidative status in plasma samples: (i) total antioxidant capacity as measured by the OXY-adsorbent test (Diacron International, Grosseto, Italy) and (ii) hydroperoxide concentration as measured by the d-ROM test (Diacron International, Grosseto, Italy). The OXY-adsorbent test quantifies the ability of plasma to oppose the massive oxidative action of hypochlorous acid through different antioxidant compounds present in plasma (Costantini, 2011). High OXY values have been described as being related to high survival probability in birds (Geiger *et al.*, 2012; Saino *et al.*, 2011). The relationship between OXY values and reproductive performance is more ambiguous (Stier *et al.*, 2012) presumably depending on how species prioritize reproduction over other life functions (Beaulieu *et al.*, 2015). The d-ROM test measures hydroperoxides, which are derived from the oxidation of fatty acids, proteins and nucleic acids and can promote cell death (Costantini, 2016). Accordingly, high plasma hydroperoxide concentrations have been associated with decreased survival probability, while they have been associated with both lower and higher reproductive performance (Costantini and Dell’Omo, 2015; Herborn *et al.*, 2015; Stier *et al.*, 2012). To measure OXY and ROM values, we followed protocols described in Beaulieu *et al.* (2010) except that spectrophotometric readings were conducted at 510 nm instead of 490 nm, as in Beaulieu *et al.* (2010). Intra- and inter-assay coefficients of variation for both tests were between 5% and 10%.

### Statistical analyses

#### (a) Inter-annual variation in regional conditions

We first examined how temperatures varied across years in the region where colonies were located. Minimal and maximal regional temperatures showed the same trends in each year. To minimize the number of regional temperature parameters reflecting the thermal conditions experienced by bats across years, we restricted our analyses to thermal anomalies averaged for minimal and maximal temperatures, which potentially reflect thermal perturbations for bats. To examine inter-annual variation in temperature anomalies, we conducted general linear models with year as a fixed factor. These analyses were conducted (i) for days during the sampling period (time between the first and the last blood sampling within each year: 12 ± 2 days (mean ± SD)) and (ii) for days during the two weeks preceding the sampling period. We chose these two time windows to examine whether oxidative markers measured at a given time point reflected current or previous thermal conditions (see further: *Relationship between environmental conditions and physiological markers*). For previous conditions, we considered two weeks preceding blood sampling, as plasma is likely to be completely renewed in small vertebrates with high metabolism like bats over longer periods of time (even though this information is lacking for *M. myotis* specifically; Hobson and Clark, 1993; Tsahar *et al.*, 2008). Plasma parameters are therefore unlikely to reflect environmental conditions occurring more than two weeks prior to blood sampling.

#### (b) Inter-colony comparison in local thermal and vegetation conditions

We examined whether colonies differed in their local thermal and vegetation conditions by using general linear mixed models with day LST, night LST, NDVI or EVI values as dependent variables, satellite measurement date (and the time of the day when Modis made LST measurements) as random factors, and year and colony as fixed factors. Because of the low temporal resolution of LST measurements, we primarily used the analyses on local thermal conditions to compare colonies (as all colonies were measured at the same time) and not to compare years. For the same reason, we did not distinguish LST data collected before and during the sampling period. More frequent measurements made by the nearby weather station were used to examine inter-annual variation in thermal conditions with greater precision (see above). Finally, we examined whether night LSTs were related to vegetation indices measured in the same 10-km perimeter by conducting general linear mixed models with NDVI or EVI values as dependent variables, colony and date as random factors and night LST as a covariate.

#### (c) Inter-annual and inter-colony variation in oxidative markers and body mass

Inter-annual variation in oxidative markers and body mass were analysed with general linear mixed models with plasma antioxidant capacity, hydroperoxide concentration or body mass, as dependent variables, individual as a random factor (some microchipped individuals were repeatedly measured across years), and year and sampling colony as fixed factors. To control for potential linear and quadratic effects of sampling time on oxidative markers (as already found in some bird species; van de Crommenacker *et al.*, 2011; Jenni-Eiermann *et al.*, 2014), sampling time (calculated as the number of minutes between sunset and blood collection) and sampling time^2^ (to test for quadratic effects) were first added to the models as covariates, and removed afterwards when not significant. For these statistical analyses, we only considered adult females, for which we had the largest sample size (183 individuals). Because the resulting sample size in 2016 was still quite low (Table 2), we repeated our statistical tests with and without considering data collected during this particular year to test the robustness of our results.

**Table 2:** Number of adult female *M. myotis* bats sampled in each year and colony in our study

	2014	2015	2016	2017	2018	Total
Béganne	NA	11	NA	14	19	44
Férel	15	16	4	8	12	55
Noyal-Muzillac	11	9	2	4	13	39
La Roche-Bernard	7	11	9	12	6	45
Total	33	47	15	38	50	183

The effects of retention duration on adult females from Saint-Nolff was also examined with a general linear mixed model, with plasma antioxidant capacity or hydroperoxide concentration as dependent variables, individual as a random factor and blood sampling number (first and second) as repeated fixed factors. We also examined if retention duration correlated with temporal variation in oxidative markers (%) using Spearman correlations.

#### (d) Relationship between environmental conditions and physiological markers

To examine whether physiological markers were directly related to regional and local environmental conditions irrespective of the colony and the year of sampling, we conducted general linear mixed models with oxidative markers or body mass as dependent variables, individual nested in colony as a random factor and temperature anomalies on the day of sampling, temperature anomalies averaged over the two weeks before the day of sampling, day LST, night LST, NDVI and EVI values as covariates. Day LST, night LST, NDVI and EVI values were averaged for a given colony in a given year. To increase the representativity of the few measurements of local conditions available (due to the 8-day temporal resolution of the satellite and because we only considered cloud-free images), we considered values measured before, during, but also slightly after the sampling period. For instance, even though three LST measurements occurred slightly after the sampling period (2–6 days later) in 2014, 2015 and 2018, we still used them to calculate average values, as LST values are averaged over an 8-day period and therefore partly encompassed the sampling period. Similarly, one NDVI/EVI measurement occurring two days after the sampling period in 2015 was used to calculate average values, as it likely reflected vegetation processes occurring during the sampling period. The effects of a given covariate on physiological markers were examined in combination with other covariates in statistical models only when variance inflation factors (VIFs) were < 3 (Dormann *et al.*, 2013). To examine the direction of the relationship between a given environmental parameter and a given physiological parameter, we conducted partial correlations between both parameters, which were corrected for all other covariates with a VIF < 3. Covariates are listed in Table 4.

#### (e) Specifications on statistical models

All two-way interactions were initially included in all models. Only models for NDVI and EVI did not include the interaction between year and colony, as we had only one to three data points per colony per year (due to the 8-day temporal resolution of the satellite and because we only considered cloud-free images). Interactions were removed from initial models using a backward procedure when they were non-significant (starting with the interaction with the highest *P*-value) and when the AIC value subsequently decreased by at least two units (Murtaugh, 2009). The normality of the residuals was tested using Shapiro–Wilk tests, and multiple comparisons were examined using Benjamini–Hochberg corrections after ordering and ranking uncorrected *P*-values. LSD (least-square differences) uncorrected *P*-values < (i x m)/Q reflected significant differences between groups (i: individual *P*-value’s rank; m: number of comparisons; Q: false discovery rate = 5%; Benjamini and Hochberg, 1995; Thissen *et al.*, 2002). All statistics were conducted in SPSS 22.00.

## Results

### (a) Inter-annual variation in regional environmental conditions

**Figure 1:**
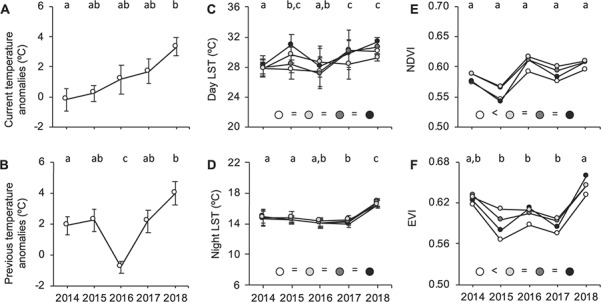
Inter-annual variation in regional and local environmental conditions. Temperature anomalies (°C) are averaged during the sampling period (A) and in the two weeks preceding blood sampling (B) between 2014 and 2018. Regional temperature anomalies were collected daily by a permanent Météo-France weather station in Saint-Nazaire-Montoir (47°18′38”N, 02°09′24”W) located 30.1 ± 7 km (mean ± SD) from our four sampling locations. Day land surface temperatures (LSTs) were measured in a 1-km radius around each bat colony (C), night LSTs were measured in a 10-km radius around each colony (D), NDVI (Normalized Difference Vegetation Index; E) and EVI (Enhanced Vegetation Index; F) values were measured in a 10-km radius around each colony. Results of local conditions (C, D, E, F) are presented for each location where bats were captured (white: Férel; light grey: Béganne; dark grey: La Roche-Bernard; black: Noyal-Muzillac) across years from 2014 to 2018. Inter-colony differences irrespective of years are represented with coloured circles at the bottom of each graph. Results are presented as mean ± SE for temperature anomalies and LST (annual means or single NDVI and EVI values are presented for each colony, as we only had one to three data points per colony per year). Years with different letters at the top of each graph significantly differ.

During the period of blood collection, thermal conditions differed between years (*F*_4, 56_ = 2.59, *P* = 0.046) with temperature anomalies increasing with successive years, becoming 3.5°C higher in 2018 than in 2014 (Fig. 1A). In the two weeks preceding blood sampling, thermal conditions also differed between years (*F*_4, 65_ = 7.01, *P* < 0.001), as all years except 2016 were warmer than normal (+ 2.6 ± 0.4°C warmer than in the period 1981–2010; Fig. 1B).

### (b) Inter-colony comparison in local thermal and vegetation conditions

Day and night LSTs did not differ between colonies (F_3, 50_ = 1.16, *P* = 0.34 and F_3, 48_ = 0.17, *P* = 0.91, respectively). However, similar to regional temperatures, day and night LST varied across years (F_4, 50_ = 6.86, *P* < 0.001 and F_4, 48_ = 19.25, *P* < 0.001, respectively) with higher values in 2018 for both day and night LST, while lower values were observed in 2014 for day LST and in 2017 for night LST. These inter-annual differences were similar across colonies (F_12, 50_ = 0.55, *P* = 0.88 and F_12, 48_ = 0.23, *P* = 0.99, respectively; Fig. 1C,D).

**Figure 2:**
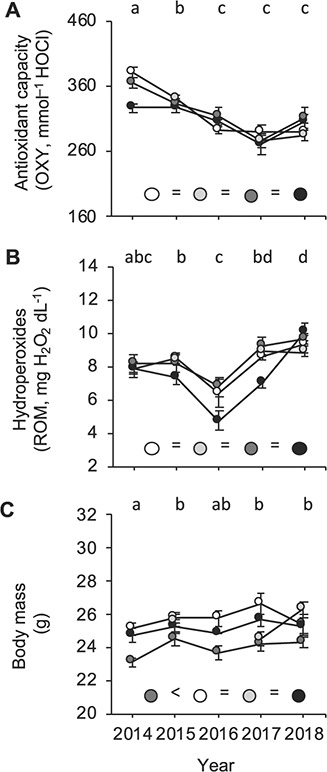
Plasma antioxidant capacity (OXY, mmol^−1^ HOCl; A), plasma hydroperoxides (ROM, mg H_2_O_2_ dL^−1^; B) and body mass (g; C), measured in early July each year from 2014 to 2018 in adult female *M. myotis* bats. Results are presented for each location where bats were captured (white: Férel; light grey: Béganne; dark grey: La Roche-Bernard; black: Noyal-Muzillac). Years with different letters at the top of each graph significantly differ and inter-colony differences irrespective of years are represented by coloured circles at the bottom of each graph. Results are presented as mean ± SE.

Within the foraging range of bats, NDVI and EVI values varied across colonies (F_3, 21_ = 9.50, *P* < 0.001 and F_3, 21_ = 5.34, *P* = 0.006, respectively) with values being lower around Férel than around other colonies (Fig. 1E,F). Vegetation indices also varied across years but only for EVI values (NDVI values: F_4, 3_ = 1.19, *P* = 0.46; EVI values: F_4, 3_ = 27.01, *P* = 0.005), with EVI values being lower in 2015, 2016 and 2017 and higher in 2018 (Fig. 2F).

Irrespective of colonies and years, we found positive relationships between vegetation indices and night LST measured in the same 10-km perimeters (NDVI: F_1, 22_ = 8.08, *P* = 0.009, B = 0.004; EVI: F_1, 24_ = 40.94, *P* < 0.001, B = 0.007, respectively).

### (c) Inter-annual and inter-colony variation in oxidative markers and body mass

Both markers of oxidative status significantly varied across years (Table 3). However, each oxidative marker followed distinct temporal patterns. Indeed, bats showed antioxidant values that were higher in 2014 than in the following years, with lowest values in 2017 (although not significantly different from 2016 and 2018; Fig. 2A). In contrast, hydroperoxide concentrations were lowest in 2016 (Fig. 2B). Moreover, inter-annual changes in oxidative status differed in amplitude depending on colony, as indicated by a significant interaction between colony and year (Table 3). This appears to be because annual variation in antioxidant capacity was less pronounced in bats from Noyal-Muzillac than in bats from other colonies (Fig. 2A), while their annual variation in oxidative damage was more pronounced (Fig. 2B). To further examine whether bats from Noyal-Muzillac were responsible for the significant interaction between colony and year, we removed this colony from our models. By considering the three remaining colonies, we found no significant interaction anymore between colony and year (antioxidant capacity: *F*_6, 131_ = 1.02, *P* = 0.42; hydroperoxides: *F*_6, 116_ = 0.67, *P* = 0.68), suggesting that the significant interaction between colony and year that we previously found was indeed due to bats from Noyal-Muzillac. Finally, colony identity also affected variation in oxidative damage during sampling time (Table 3). Indeed, by repeating our model for each colony separately, we found that hydroperoxide levels did not show any effects of sampling time in bats from Férel and La Roche-Bernard (all *P* > 0.41), while they followed a U-curve and an inverse U-curve in bats from Béganne and Noyal-Muzillac, respectively (sampling time^2^: *F*_1, 39_ = 5.99, *P* = 0.019 and *F*_1, 32_ = 7.46, *P* = 0.010, respectively; Fig. S1). All statistical results on antioxidant capacity and hydroperoxide levels remained unaffected after excluding data from 2016, thereby suggesting that although this year had low sample sizes, it did not disproportionally affect our results (see Supplementary Material). Moreover, retention duration did not affect nor correlate with antioxidant values (*F*_1, 7_ = 0.06, *P* = 0.82; r_s_ = 0.02, *P* = 0.96) or hydroperoxide concentrations (*F*_1, 7_ = 0.14, *P* = 0.72; r_s_ = −0.04, *P* = 0.93).

**Table 3:** Results of final statistical models examining the effects of year, colony and sampling time on body mass and oxidative markers [plasma hydroperoxides (ROM) and antioxidant capacity (OXY)] in adult female *M. myotis*. Interactions, sampling time and sampling time^2^ were removed from the final models when non-significant

	Hydroperoxides (ROM, mg H_2_O_2_ dL^−1^)	Antioxidant capacity (OXY, mmol^−1^ HOCl)	Body mass (g)
Year	**F**_**4, 150**_ **= 14.39, P < 0.001**	**F**_**4, 165**_ **= 27.14, P < 0.001**	***F***_**4, 93**_ **= 2.79, *P* = 0.031**
Colony	F_3, 155_ = 0.80, P = 0.50	F_3, 165_ = 1.11, P = 0.35	***F***_**3, 163**_ **= 9.01, *P* < 0.001**
Sampling time	F_1, 155_ = 0.38, P = 0.54	-	-
Sampling time^2^	F_1, 155_ = 0.10, P = 0.75	-	-
Year*colony	**F**_**10, 146**_ **= 2.09, P = 0.029**	**F**_**10, 165**_ **= 1.99, P = 0.037**	***F***_**10, 92**_ **= 2.72, *P* = 0.006**
Colony* sampling time	F_3, 155_ = 1.91, P = 0.13	-	-
Colony* sampling time^2^	**F**_**3, 155**_ **= 3.11, P = 0.028**	-	-

Body mass significantly varied across years with bats being lighter in 2014 than in 2015, 2017 and 2018. Bats from La Roche-Bernard were also consistently lighter than bats from other colonies (La Roche Bernard: 24.0 ± 0.2 g, other colonies: 25.5 ± 0.1 g; post hoc tests: all *P*-values > uncorrected *P*-values; Fig. 2C). Finally, inter-annual variation in body mass differed between colonies. Indeed, by repeating our model for each colony separately, we found that body mass did not differ across years in Noyal-Muzillac or La Roche-Bernard (*F*_4, 3_ = 0.87, *P* = 0.57 and *F*_4, 30_ = 2.00, *P* = 0.12, respectively) while it did in Férel and Béganne (*F*_4, 12_ = 10.46, *P* = 0.001 and *F*_2, 27_ = 9.13, *P* = 0.001, respectively). However, both colonies showed opposite temporal trends, as bats were heaviest in 2017 in Férel (post hoc tests: all *P* values > uncorrected *P*-values) while they were lightest in 2017 in Béganne (post hoc tests: all *P* values > uncorrected *P*-values; Fig. 2C).

### (d) Relationship between environmental conditions and physiological markers

**Figure 3:**
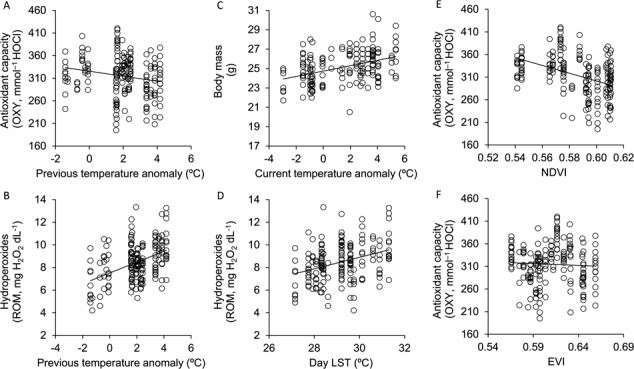
Significant relationships between environmental conditions (x-axis) and physiological parameters (y-axis). Raw data are presented. Statistical results are presented in Table 4.

The temperature anomalies occurring in the two weeks preceding blood sampling negatively correlated with the antioxidant capacity of bats and positively correlated with their hydroperoxide levels (Table 4, Fig. 3). Similarly, day LST values tended to be negatively correlated with the antioxidant capacity of bats and were positively correlated with hydroperoxide levels. In contrast, night LST values did not correlate with any of the physiological parameters we considered. The temperature anomalies occurring on the day of blood sampling did not correlate either with oxidative markers, but they positively correlated with the body mass of bats (Table 4, Fig. 3). Finally, NDVI and EVI negatively and positively correlated, respectively, with the antioxidant capacity of bats (Table 4, Fig. 3). All statistical results on the relationship between environmental conditions and physiological markers are summarized in Table 1.

**Table 4:** Results of statistical models examining the effects of each environmental condition considered in our study (T°: temperature, LST: Land Surface Temperature, NDVI: Normalized Difference Vegetation Index, EVI: Enhanced Vegetation Index) on physiological parameters (antioxidant capacity, hydroperoxides, body mass) in adult female *M. myotis*. Significant effects are highlighted in boldface, and the covariates included in each model are indicated in the last column

	Antioxidant capacity	Hydroperoxides	Body mass	Covariates
Previous T° anomalies	**F**_**1, 177**_ **= 4.95** ***P* = 0.027** **r = −0.17**	**F**_**1, 167**_ **= 11.23 *P* = 0.001** **r = 0.25**	F_1, 106_ = 1.56 *P* = 0.22 r = 0.17	Current T° anomalies, day & night LST, NDVI
Current T° anomalies	F_1, 179_ = 1.86 *P* = 0.17 r = −0.10	F_1, 177_ = 0.14 *P* = 0.71 r = −0.04	**F**_**1, 146**_ **= 40.12 *P* < 0.001** **r = 0.41**	Day LST, NDVI
Day LST	F_1, 179_ = 3.67 *P* = 0.057 r = −0.14	**F**_**1, 177**_ **= 14.78 *P* < 0.001** **r = 0.28**	F_1, 165_ = 3.77 *P* = 0.054 r = −0.18	Current T° anomalies, NDVI
Night LST	F_1, 176_ = 0.004 *P* = 0.95 r = 0.005	F_1, 167_ = 0.06 *P* = 0.80 r = 0.01	F_1, 128_ = 0.05 *P* = 0.82 r = −0.002	Previous & current T° anomalies, day LST, NDVI, EVI
NDVI	**F**_**1, 178**_ **= 105.45 *P* < 0.001** **r = −0.61**	F_1, 169_ = 1.06 *P* = 0.31 r = 0.09	F_1, 117_ = 0.03 *P* = 0.87 r = 0.00	Current T° anomalies, day LST, EVI
EVI	**F**_**1, 178**_ **= 48.55 *P* < 0.001** **r = 0.46**	F_1, 159_ = 1.65 *P* = 0.201 r = 0.09	F_1, 108_ = 0.62 *P* = 0.43 r = −0.08	Current T° anomalies, day LST, NDVI

## Discussion

As expected, plasma oxidative markers varied in free-ranging greater mouse-eared bats across years in parallel with thermal conditions: antioxidant capacity decreased while hydroperoxide concentrations increased with higher temperature anomalies occurring in the two weeks before the sampling period but not with temperature anomalies occurring on the day of sampling. As the amplitude of temperature anomalies was similar before and during the sampling period (ca. 4°C), these results suggest that oxidative markers reflected recent past thermal conditions (< two weeks) and not current thermal conditions. This further suggests that plasma antioxidant capacity needs time to reflect new thermal conditions and that hydroperoxides are not immediately eliminated but rather accumulate in plasma presumably because of their stability (Frei *et al.*, 1988). In support of this hypothesis, plasma hydroperoxides do not immediately vary in captive zebra (*Taeniopygia guttata*) and Gouldian (*Erythrura gouldiae*) finches exposed to different temperatures but may only increase in Gouldian finches after several weeks following exposure (Beamonte-Barrientos and Verhulst, 2013; Beaulieu *et al.*, 2014; Fragueira *et al.*, 2019).

In our study, the fact that the plasma antioxidant capacity of bats decreased across years suggests that they became progressively less oxidatively challenged with time. Relaxed oxidative conditions with increasing temperature may explain why increasing summer temperatures were recently found to be negatively correlated with telomere attrition in *M. myotis* bats from the same colonies (Foley *et al.*, 2020), as telomeres appear to be highly sensitive to the damaging effects of ROS (von Zglinicki, 2002). Lower antioxidant capacity and telomere attrition may be due to temperature increases over successive years, which exposed bats to temperatures closer to their thermoneutral zone (28–> 37°C), thereby reducing thermoregulatory costs. Similarly, Gouldian finches exposed to a temperature within their thermoneutral zone after being exposed to a temperature below their lower critical temperature decrease their plasma antioxidant capacity (Beaulieu *et al.*, 2014). Reduced investment into self-maintenance mechanisms, such as thermoregulation and antioxidant defences, may have allowed female bats experiencing higher temperatures to invest more strongly into reproduction. Accordingly, a previous study found that higher temperatures (up to 24°C) enhanced the development of *M. myotis* pups presumably because of higher maternal investment and because mothers and pups spend less time in torpor under such conditions (Zahn, 1999). A lower use of daily torpor may also explain the lower antioxidant capacity that we observed with higher temperatures (Wojciechowski *et al.*, 2007). Indeed, arousing from torpor is costly and increases ROS production, in response to which bats can activate antioxidant defences (Filho *et al.*, 2007). This hypothesis is supported by the fact that the antioxidant capacity of bats tended to be negatively related to day LST (measured when bats were roosting) while it was unrelated to night LST (measured when bats were foraging). Thermal conditions likely affected the antioxidant capacity of foraging bats also indirectly by acting on vegetation quality and insect abundance. Indeed, we found that night LSTs were positively correlated with vegetation indices, which in turn were correlated with the antioxidant capacity of bats. The fact that only EVI values were positively correlated with the antioxidant capacity of bats is likely due to the fact that EVI values better predict insect abundance in western France (Lafage *et al.*, 2014). The reasons why NDVI values negatively correlated with the antioxidant capacity of bats are unclear, but it may be speculated that higher NDVI values are related to a more complex and denser habitat where insects are more difficult to catch, thereby increasing the foraging costs and decreasing the antioxidant defences of these bats.

Because the amplitude of temperature anomalies likely exposed bats to temperatures closer to thermoneutrality, bats exposed to higher temperatures were expected to show lower levels of oxidative damage. In contrast, we found that temperature anomalies occurring within the two weeks preceding blood sampling and day LST were positively related to hydroperoxide levels, suggesting that bats’ maintenance was negatively affected by higher temperatures. Importantly, inter-annual differences in hydroperoxide levels did not mirror inter-annual differences in body mass, suggesting that higher values of hydroperoxide levels were not related to higher body condition and fat reserves (Pérez-Rodríguez *et al.*, 2015). A potential explanation for the counter-intuitive increase in oxidative damage that we observed is that slightly warmer conditions associated with lower thermoregulatory and foraging costs may have allowed bats to invest more into reproduction, which may result in higher plasma hydroperoxide levels (Stier *et al.*, 2012). Indeed, higher temperatures up to 24°C have been shown to favour the development of *M. myotis* pups presumably because of higher maternal investment (Zahn, 1999). However, bats appear to be able to increase reproductive performance without deteriorating self-maintenance, as shown in lesser horseshoe bats (*Rhinolophus hipposideros*; Jan *et al.*, 2019). If these results hold true for female *M. myotis*, another explanation for the higher hydroperoxide levels that we observed with higher temperatures in our study is that slightly higher temperatures outside the roost of bats (as measured here) resulted in much higher temperatures inside the roost exceeding the upper critical temperature of bats and increasing ROS production (Johnson and Lacki, 2014). The fact that only day LST, and not night LST, positively correlated with hydroperoxide levels in our study supports this hypothesis. In that case, the decreased antioxidant capacity that we observed with increasing temperatures would reflect a higher demand for and a higher use of antioxidant defences to counteract higher ROS production under hot roosting conditions (in contrast to the hypothesis described in the previous paragraph). However, this antioxidant response only appears partial, as oxidative damage still increased with higher day LST values. The monitoring of the reproductive performance of bats in parallel with a precise monitoring of the thermal conditions inside roosts would be necessary to disentangle the two explanations potentially underlying the higher hydroperoxides levels that we observed with increasing temperatures (i.e. increased reproductive performance vs. increased thermoregulatory costs).

In all the colonies considered in our study, oxidative markers varied in the same direction across years. However, the magnitude of these changes differed between colonies, with one colony (Noyal-Muzillac) showing smaller changes in antioxidant capacity and higher changes in hydroperoxide levels across years than other colonies. It is unlikely that these bats experienced local thermal and foraging conditions that differed from those experienced by bats from other colonies, as their condition (as assessed by their body mass), habitat (as assessed by vegetation indices) and local thermal conditions (as assessed by LST) were similar to those of bats from other colonies. However, as their body condition appeared to be more stable across years than that of bats from other colonies, it may be hypothesized that these bats experience more stable feeding conditions than most bats in other colonies (even though vegetation indices were not more stable across years for this colony). Moreover, their oxidative status appears to show higher plasticity compared to other colonies, as they were among the only two colonies showing effects of sampling time on hydroperoxide levels (even though both colonies show opposite temporal patterns, and even though such differences between colonies may be due to unavoidable small differences in the timing of bat sampling in each colony). Overall and irrespective of the underlying mechanisms, which currently remain unclear, our results suggest that the regulation of the oxidative status of bats is colony specific.

Our study highlights the difficulty of adopting physiological parameters in conservation studies, as we were limited by (i) the number of individuals to monitor year after year in each of the four colonies we followed, (ii) the number of oxidative markers we could measure in small plasma samples collected in small mammals and (iii) the number of tissues accessible in a protected species (here only plasma). Our results add another layer of complexity to this list, as they suggest that the regulation of oxidative markers is colony specific. Indeed, the interaction between colony and year that we found for both oxidative markers suggests variable plasticity in oxidative status in relation to environmental conditions. Assuming that the plasticity in oxidative status in relation to temperature indeed differs between colonies and is not due to subtle unnoticed differences in environmental conditions, it would be interesting to examine whether such differences between colonies affect the fitness of individuals within each colony. Indeed even though our study suggests that oxidative markers can be used to track *a posteriori* changes in the thermal conditions of animals in their natural habitat, oxidative markers also need to have a predictive value to be useful in conservation programs (Beaulieu and Costantini, 2014). Towards this end, more data on the relationship between oxidative markers and fitness components or demographic processes are needed. For instance, in *Pygoscelis* penguins, colonies experiencing demographic growth show higher levels of antioxidant defences than colonies with a decreasing trend (Beaulieu *et al.*, 2013). In our study, we do not know the overall performance of bats in relation to their physiological response to thermal conditions, at times making the interpretation of our results challenging (e.g. increased oxidative damage with increasing temperatures). Filling this gap would allow conservation practitioners to define the level of threat that thermal conditions may represent for each colony and which specific colony may actually need conservation measures. Oxidative markers should therefore complement population monitoring to aid conservation practitioners in implementing the most appropriate conservation measures at the local scale.

## Funding

E.C.T. was supported by a research grant of the European Research Council Research (ERC-2012-StG311000). M.B. and S.J.P. were funded by the University of Greifswald and associated to the research training group RESPONSE when biological samples were collected and measured.

## Supplementary Material

Supplementary_material_coaa083
