# Supplementary material for: Timescale and colony-dependent relationships between environmental conditions and plasma oxidative markers in a long-lived bat species
Source: Conserv Physiol. 2020 Sep 14;8(1):coaa083. doi: 10.1093/conphys/coaa083 (PMC7605240; doi:10.1093/conphys/coaa083)
Supplement: Supplementary_material_coaa083 [file supplementary_material_coaa083.docx]

**Table S1.** Results of final statistical models examining the effects of year, colony (excluding Noyal-Muzillac) and sampling time on body mass and oxidative markers (plasma hydroperoxides and antioxidant capacity) in adult female *Myotis myotis*.

|  | Hydroperoxides  (ROM, mg H_2_O_2_ dL^-1^) | Antioxidant capacity  (OXY, mmol^-1^ HOCl) | Body mass  (g) |
| --- | --- | --- | --- |
| Year | **F_3, 143_ = 10.19, P < 0.001** | **F_3, 153_= 34.79, P < 0.001** | ***F*_3, 110_ = 3.38, *P* = 0.021** |
| Colony | F_3, 143_ = 0.67, P = 0.58 | F_3, 153_ = 2.22, P = 0.09 | ***F*_3, 148_ = 8.20, *P* < 0.001** |
| Sampling time | F_1, 143_ = 0.02, P = 0.89 | - | - |
| Sampling time^2^ | F_1, 143_ = 0.01, P = 0.92 | - | - |
| Year*colony | **F_8, 143_ = 2.09, P = 0.029** | **F_8, 153_ = 2.22, P = 0.029** | ***F*_8, 100_ = 2.92, *P* = 0.006** |
| Colony*sampling time | F_3, 143_ = 1.48, P = 0.22 | - | - |
| Colony*sampling time^2^ | F_3, 143_ = 2.58, P = 0.056 | - | - |

**Figure S1** Relationships between sampling time (in minutes after sunset) and hydroperoxides (white: Férel, light grey: Béganne, dark grey: La Roche-Bernard, black: Noyal-Muzillac). Regression lines are represented for colonies where hydroperoxide levels depended on sampling time.
